# Prevalence and Molecular Characterization of *Rickettsia* spp. from Wild Small Mammals in Public Parks and Urban Areas of Bangkok Metropolitan, Thailand

**DOI:** 10.3390/tropicalmed6040199

**Published:** 2021-11-11

**Authors:** Artharee Rungrojn, Kittipong Chaisiri, Yossapong Paladsing, Serge Morand, Jiraphan Junjhon, Stuart D. Blacksell, Peeraya Ekchariyawat

**Affiliations:** 1 Department of Microbiology, Faculty of Public Health, Mahidol University, Bangkok 10400, Thailand; Arthareer@yahoo.com (A.R.); jiraphan.juh@mahidol.ac.th (J.J.); 2 Department of Helminthology, Faculty of Tropical Medicine, Mahidol University, Bangkok 10400, Thailand; kittipong.cha@mahidol.ac.th (K.C.); nook5508077@gmail.com (Y.P.); 3 CNRS ISEM-CIRAD ASTRE, Faculty of Veterinary Technology, Kasetsart University, Bangkok 10220, Thailand; serge.morand@umontpellier.fr; 4 Mahidol-Oxford Tropical Medicine Research Unit, Faculty of Tropical Medicine, Mahidol University, Bangkok 10400, Thailand; Stuart@tropmedres.ac; 5 Centre for Tropical Medicine and Global Health, Nuffield Department of Clinical Medicine, University of Oxford, Old Road Campus, Roosevelt Drive, Oxford OX3 7FZ, UK

**Keywords:** *Rickettsia typhi*, *Rickettsia felis*, small mammals, rodents, public parks, urban, Bangkok

## Abstract

Rural areas usually show a higher prevalence of rickettsial infection than urban areas. However, information on the rickettsial infection status in urban settings (e.g., built-up areas and city parks) is still limited, particularly in the Bangkok metropolitan area. In this study, we performed a molecular rickettsial survey of spleen samples of small mammals caught in public parks and built-up areas of Bangkok. Out of 198 samples, the *Rattus rattus* complex was found to be most prevalent. The amplification of rickettsial *gltA* fragment gene (338 bp) by nested PCR assay revealed positive results in four samples, yielding a low prevalence of infection of 2.02%. DNA sequencing results confirmed that three samples were matched with *Rickettsia typhi*, and one was identified as *R. felis*. It is noteworthy that this is the first report of the occurrence of *R. felis* DNA in rodents in Southeast Asia.

## 1. Introduction

*Rickettsia* (order Rickettsiales; family Rickettsiaceae) are small, Gram-negative, obligate intracellular bacteria [1,2]. *Rickettsia* spp. are best known as vector-borne pathogens and include blood-sucking arthropod vectors, such as lice, ticks, fleas, and mites [1]. These pathogens infect a wide range of vertebrate hosts, such as small mammals and humans. The genus *Rickettsia* can be classified into four groups—a spotted fever group (SFG), typhus group (TG), transitional group, and non-pathogenic ancestral group [3].

Murine typhus, a flea-borne acute febrile illness caused by the bacteria *R. typhi* of TG rickettsiae, has been reported worldwide. The mortality rate of murine typhus is low (1% of reported cases) [4]; however, it remains one of the most prevalent human rickettsial infections throughout the world [5,6]. The infection initiates pathological changes leading to an increase in vascular permeability; rash; and, in severe illness, interstitial pneumonia, meningoencephalitis, and multi-organ failure [7,8,9]. Animals that have been recognized as reservoir hosts include dogs, cats [10,11], wild and domestic ruminants, and other wildlife [12] (e.g., reptiles [13], birds [14,15], boars [16], and rodents).

*R. felis*, a causative agent of cat-flea typhus infection, one of the flea-borne rickettsioses, belongs to the transitional group of the genus *Rickettsia*. Cat fleas (*Ctenocephalides felis*) play an important role in maintaining *R. felis*. Additionally, lice, ticks, and mosquitoes are known to serve as potential vectors, but the transmission mechanism of temporary infections is still not clear [17]. The transmission of *R. felis* to vertebrate hosts mostly occurs through flea bites or flea feces via broken skin [18,19]. In Thailand, the first case report of *R. felis* infection in a human was documented in 2003 [20]. There has also been molecular evidence of *R. felis* infection in two patients from Chiang Rai Province; both of these patients had a common history of contact with animals and probably the flea vector [21]. 

Rodents, particularly rats and mice, are one of the most diversified groups in the order Mammalia [22]. They live in a variety of terrestrial natural habitats, including human-made environments. This is particularly important for rodent-borne pathogens that are zoonotic emerging or remerging diseases [22]. Public parks or urban gardens are vitally important for establishing and maintaining the quality of life in a community, ensuring the health of families and youth, and contributing to the economic and environmental well-being of a community and nearby areas. Many people use public parks for social activities and education. However, there are also many people who enter public parks due to professional activities, such as security guards and gardeners. Many animals also share the same park space as their habitat for foraging, breeding, and residence. There is a significant potential for zoonotic disease transmission in urban settings, such as green areas and public parks, which is concerning [23,24,25]. To date, several studies have attempted to assess the occurrence of *Rickettsia* spp. in reservoir animals living in recreational city parks—e.g., in Arkansas, United States [26]; Rome, Italy [27]; and Bavaria, Germany [28]. However, information on the existence of *Rickettsia* spp. in the urban areas and public parks of Bangkok metropolitan area, Thailand, is limited. 

This study aimed to investigate the prevalence and characterize rickettsiae isolated from small mammals in Bangkok metropolitan area, particularly from green areas, such as public parks. This will provide basic information on the occurrence of rickettsial infection in Bangkok metropolitan area, benefitting disease prevention and control strategies as well as raising awareness about the risk of disease transmission from animals that live closely alongside humans in urban settings.

## 2. Materials and Methods

### 2.1. Study Area and Small Mammal Trapping

**Figure 1:**
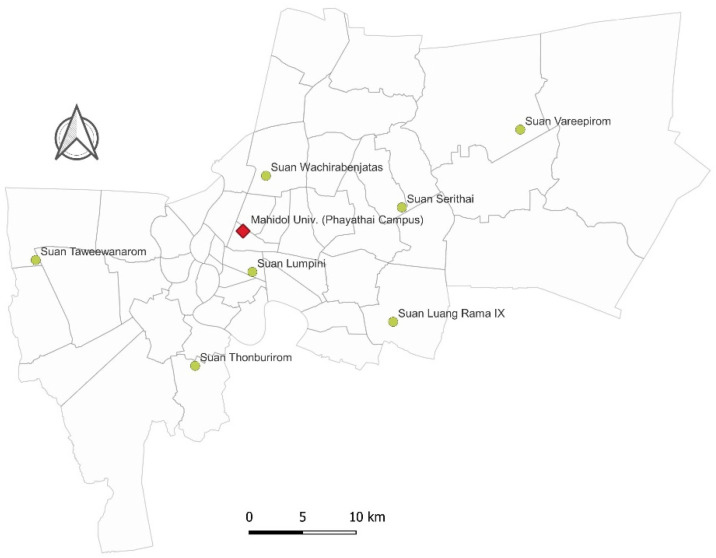
The distribution of public parks and build up areas within the Bangkok metropolitan area. The green color indicates the public parks used for animal sampling. The red color indicates the urban area trapping site located at Mahidol University (Phayathai Campus).

Between September and December 2018, small mammals (rodents and shrews) were trapped at 7 public parks located in the Bangkok metropolitan area—Suan Luang Rama IX, Suan Lumpini, Suan Serithai, Suan Taweevanarom, Suan Thonburirom, Suan Vareepirom, and Suan Wachirabenjatas (see details of the trapping protocol used in Paladsing et al. [29]). In addition, animals were also captured at the urban site of Mahidol University, Phayathai Campus, Bangkok (Figure 1). Animals were humanely euthanized using isoflurane inhalation and subsequently speciated before specimens were collected. The protocols used for animal handling and specimen collection were approved by the Faculty of Tropical Medicine-Animal Care and Use Committee, Mahidol University (FTM-ACUC), under document number FTM-ACUC 016/2018E, and Mahidol University-Institute Animal Care and Use Committee, Mahidol University (MU-IACUC), under document number MU-IACUC 2021/009.

### 2.2. Nucleic Acid Extraction and Detection of Rickettsial DNA

Spleen tissues were collected from the small mammals, preserved in RNAlater solution (Sigma–Aldrich), and stored at −20 °C. Genomic DNA was extracted from the spleen tissues using the Genomic DNA mini kit (tissue) (Geneaid, Taiwan); we followed the manufacturer’s instructions for all procedures. *R. typhi* genomic DNA was obtained from the Lao-Oxford-Mahosot Hospital-Wellcome Trust Research Unit (LOMWRU) and used as a positive control. All samples were tested for rickettsia using nested PCR assays. PCR amplification of 382 bp of *gltA* was carried out using primers previously described by Kuo et al. (2015): RpCS.877p and RpCS.1258n for the 1st round of PCR, followed by the amplification of 338 bp of *gltA* using RpCS.896 and RpCS.1233n primers in the 2nd round of PCR [30]. The PCR conditions used were as follows: PCR was performed in 20 μL of sample, consisting of 0.4 µM of each *gltA* forward and reverse primer, 200 µM of each deoxy nucleotide triphosphate, 1.25 U of GoTaq^®^ DNA polymerase (Promega Corp., Madison, WI, USA), 1× PCR buffer, and 1 µL of DNA template. All samples were tested in two replicates. DNA fragments were pre-denatured at 95 °C for 10 min, followed by 40 cycles of denaturation at 95 °C for 30 s, annealing at 45 °C for 30 s, extension at 72 °C for 55 s, and post extension at 72 °C for 10 min on an MJ mini thermal cycler, Biorad. Gel electrophoresis was performed with a 1.5% agarose gel stained with 2.5% RedSafe™ (iNtRON Biotechnology, Inc., Seongnam-si, Korea), which was used for DNA product separation and visualized under UV fluorescence.

### 2.3. Sequencing and Phylogenetic Analysis

PCR-positive samples were subjected for DNA sequencing (Barcode-tagged sequencing service, U2Bio Thailand). A phylogenetic tree was re-constructed with the maximum likelihood (ML) method using the MEGA 7 program on the Tamura 3-parameter model [31]. Bootstrap analysis with 1000 repetitions was performed to test the robustness of the tree branching. The nucleotide basic local alignment search tool (BLASTn) [32] was used to compare the DNA sequences obtained in this study with those on the Genbank database.

## 3. Results

### 3.1. Sampling

A total of 198 small mammals were trapped in public parks across Bangkok metropolitan area and the site at Mahidol University, Phayathai Campus. The majority of samples were collected in Suan Wachirabenjatas (27.8%), Suan Luang Rama IX (19.2%), and Mahidol University Phayathai campus (17.2%) (Table 1). Five small mammal species were collected: order Rodentia: *Ratttus exulans*, *R. norvegicus* and *R. rattus*-complex; order Scandentia: *Tupaia belangeri*; and order Eulipotyphla: *Suncus murinus*. The *R. rattus* complex was the predominant species (81.8%), followed by *T. belangeri* (9.6%), *R. exulans* (6.1%), *S. murinus* (1.5%), and *R. norvegicus* (1.0%) (Table 1).

### 3.2. PCR and DNA Sequencing

**Figure 2:**
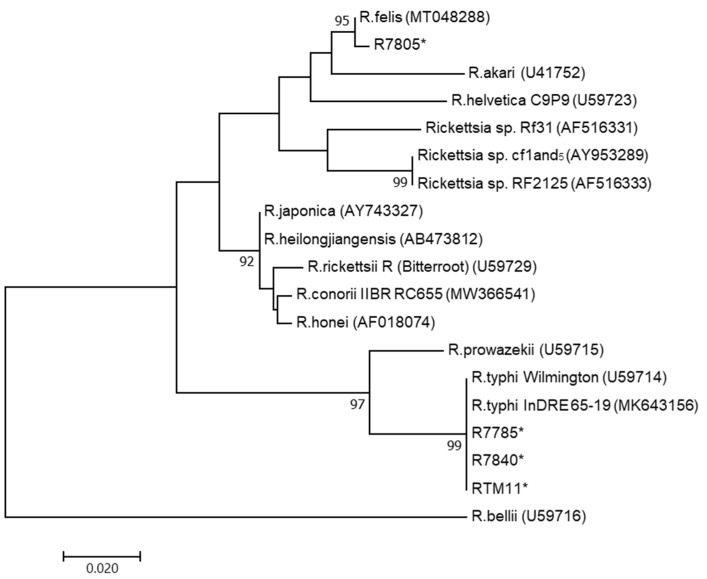
Unrooted phylogenetic reconstruction of the rickettsial *gltA* gene using the maximum likelihood method based on the Tamura 3-parameter model. The tree topology is drawn to scale, with branch lengths indicating the number of substitutions per site. Bootstrap values higher than 70% are indicated. Positive samples are denoted (*) after the sample ID (R7785, R7805, R7840, and RTM11 belong to the Rattus rattus complex).

Using the nested PCR targeting rickettsial *gltA*, four out of 198 (2.0%) samples were found to be positive and their products were sequenced to confirm their species. Three samples (R7785, R7840, RTM11) demonstrated a 99.7-100% sequence similarity to *R. typhi*: accession no. MN497621.1 and MK643156. One sample (R7805) demonstrated a 99.7% sequence similarity to *R. felis:* accession no. MT048288.1. The phylogenetic analysis of the rickettsial *gltA* gene from the positive rodents was compared with other related *Rickettsia* sequences available in GenBank (Figure 2). All these samples belonged to rodents of the *R. rattus* complex.

## 4. Discussion

In this study, we reported evidence of the molecular detection of *R. typhi* and *R. felis* in small mammals (particularly in the *R. rattus* complex, who as predominant hosts accounted for 82% of all captured animals) in public parks and urban areas across metropolitan Bangkok. The *R. rattus* complex is a synanthropic rodent species, since members of this species are pervasive and live close to humans in urban settings [33,34]. Synanthropic rodents are important vectors of zoonotic pathogens and pose a significant risk to public health [35]; they also serve as disease reservoirs and spread pathogens to humans either directly or indirectly via ectoparasite vectors, which is the case for rickettsial infections [36].

Rickettsial infections (with the exclusion of scrub typhus) in small mammals have been reported in Asian countries, with differences seen in the prevalence of various pathogens. Using molecular tools, a high rate of rickettsial infection in small mammal species was reported from a country-wide surveillance effort in Taiwan. The total prevalence of infection was up to 60.5%, with nine *Rickettsia* species identified: *R. conorii*, *R. felis*, *R. japonica*, *R. raoultii*, *R. rickettsii*, *Rickettsia* sp. IG-1, *Rickettsia* sp. TwKM01, *Rickettsia* sp. TwKM02, and *R. typhi* [30]. In the Jiangsu and Jiangxi provinces of China, the prevalence of rickettsial infections ranged from 9.23 to 13.95% in Asian house shrews (*S. murinus*), house mice (*Mus musculus*), field mice (*Apodemus agrarius*), and lesser rice field rats (*R. losea*). Several rickettsial species were also reported, including *R. heilongjiangensis*, *R. japonica*, *Rickettsia parkeri*-like strain, *R. raoultii*, *Anaplama phagocytophilum*, *Ehrlichia* sp., and Candidatus *Neoehrlichia mikurensis* [37]. In wet markets in Kuala Lumpur and Palau Pinang in Malaysia, 13.7% of the captured rats (*R. rattus diardii* and *Rattus norvegicus*) were found to be positive for *R. honei*/*R. conorii*/*R. raoultii* after a DNA sequence analysis [38].

Two studies recently reported on the seroprevalence of *R. typhi* in rodents from Thailand [39,40]. Chareonviriyaphap et al. (2014) [39] reported a 23.7% seroprevalence of *R. typhi* in wild rodents collected from 10 provinces across four regions of Thailand (central, northern, northeastern, and southern parts of Thailand). In the suburbs of Bangkok, the seroprevalence of murine typhus exposure in oriental house rats (*R. tanezumi*) ranged from 32.31 to 64.15%. It should be noted that positivity was mainly found in human-dominated habitats (i.e., residences and animal shelters) and that no murine typhus antibody positivity was reported in rats captured in agricultural fields [40].

In the present study, we reported a relatively low prevalence of rickettsial infection (2.02%) in small mammals from the Bangkok metropolitan area. A lower prevalence found by molecular detection is to be expected, as molecular evidence tends to infer active infection in the host. Seropositivity indicates past or current exposure, and thus finding a higher prevalence is not uncommon [41,42]. We also observed that the prevalence of arthropod vectors was quite low in the study sites. Of all the animal hosts analyzed in this study, we found only a small number of rat fleas on one rat captured in Suan Serithai, while two animals (*T. belangeri* from Suan Thonburirom and *R. rattus* complex from Mahidol University Phayathai campus) were found to be infested with lice. Vector-borne pathogen transmission may be exacerbated by a high abundance of disease vector. The population dynamics and survival of arthropod ectoparasites are influenced by environmental factors, including seasonal and climatic effects [43,44]; however, these effects were not addressed in our study.

The molecular detection of the occurrence of *R. felis* in a *R. rattus* complex from Bangkok public park is the first such report in Southeast Asia. To date, almost 40 arthropod species (i.e., fleas, ticks, lice, and mosquitoes) have been shown to harbor *R. felis* [17]. A number of domestic animals, including cats, dogs, and wildlife (opossums, raccoons, and rodents) have been found to be infected by *R. felis* [17]. Past publications have shown that *R. felis* has been detected in rodent species across the world, including Columbia, Mexico, Taiwan, and Zambia [30,45,46,47]. In Thailand and other countries in Southeast Asia (Indonesia, Malaysia, the Philippines, and Vietnam), *R. felis* has been detected mainly in flea samples collected from dogs, cats, and small mammals [38,39,48]. However, there are no reports of *R. felis* detection occurring directly from rodents in this region. 

Human rickettsial infection has been documented in Bangkok, but information on the infection status of mammalian reservoir hosts (particularly small mammals) in the city is limited. The molecular diagnosis of buffy coat samples of human cases with idiopathic fever confirmed that the patients were positive for *R. typhi*, *R. felis*, and *R. felis*-like sp. [11]. In the same study, *R. felis* and *R. felis*-like sp. were also detected in domestic dogs in Bangkok. Together with the results of this study, this suggests that synanthropic rodents living in the city could play a role as potential reservoirs in the epidemiology of rickettsial disease in urban areas such as Bangkok.

This study has some limitations. Firstly, *R. felis* and *R. typhi* were found to be positive by PCR and the sequencing result was only based on the gltA gene. The rickettsia typhus group should also be investigated in a further study. We note that we collected ectoparasites (i.e., mites, lice, and fleas) from captured small mammal hosts, but we did not perform rickettsial detection in those ectoparasites. The rickettsial investigation of fleas and other arthropod vectors should therefore be performed in the future to add to the epidemiological information of rickettsioses in urban areas, such as Bangkok. Additionally, further studies should include field sampling in both wet and dry seasons to determine the seasonal effects on ectoparasite abundance and *Rickettsia* infection. Moreover, information on the management practices of each public park (such as the use of chemical substances or pesticides) could also be taken into account to determine whether these may affect the population of ectoparasites and subsequently the transmission of pathogens, particularly in ornamental garden areas.

## 5. Conclusions

The current findings concerning *R. typhi* and *R. felis* in small mammals from urban areas can help to inform those responsible for the administration of Bangkok public parks and other communal areas and nearby communities of the threat of rickettsioses.

## Figures and Tables

**Table 1:** Number of small mammals trapped from different sites in this study.

Order	Family	Species	Suan Luang Rama IX	Suan Lumpini	Suan Serithai	Suan Taweevanarom	Suan Thonburirom	Suan Vareepirom	Suan Wachirabenjatas	Mahidol University Phyathai Campus	Total
Rodentia	Muridae	*R. exulans*	0	1	4	2	4	0	0	1	12 (6.1)
Rodentia	Muridae	*R. norvegicus*	0	1	0	0	0	0	0	1	2 (1.0)
Rodentia	Muridae	*R. rattus*-complex	31	2	13	11	5	23	48	29	162 (81.8)
Scandentia	Tupaiidae	*T. belangeri*	7	0	0	2	3	0	7	0	19 (9.6)
Eulipotyphla	Soricidae	*S. murinus*	0	0	0	0	0	0	0	3	3 (1.5)
Total			38 (19.2)	4 (2.0)	17 (8.6)	15 (7.6)	12 (6.1)	23 (11.6)	55 (27.8)	34 (17.2)	198

## Data Availability

Not applicable.
